# A Cultural Shift Away from Cognitive-behavioral Empathy

**DOI:** 10.7759/cureus.6175

**Published:** 2019-11-17

**Authors:** James B Fowler, Yasir R Khan, Glenn M Fischberg, Deependra Mahato

**Affiliations:** 1 Neurological Surgery, Desert Regional Medical Center, Palm Springs, USA; 2 Neurology and Neurological Surgery, Desert Regional Medical Center, Palm Springs, USA

**Keywords:** empathy, education, cognitive-behavioral, neurosurgery, medical education, patient satisfaction

## Abstract

Empathy in medicine is often neglected due to various constraints imposed on the physician. Despite empathy being proven as beneficial to the patient health and outcomes, patients remain unsatisfied with the healthcare system and usually, in turn, their physicians. To instill empathetic patient-physician relationships, medical training has for some time focused on cognitive-behavioral empathy. This is taught through cognitive and behavioral skills, with expressions such as “I understand how you feel”. Naturally, these skills are often forced and feel disingenuous. Hence, a cultural shift in medicine is required to effectively communicate the importance of empathy: a shift that cultivates altruistic properties most healthcare professionals bring to medicine in the first place.

## Introduction and background

There is a growing division in medicine, and it constitutes a divide between patients and their physicians. This divide fundamentally stems from the patient dissatisfaction with today’s healthcare system and a lack of clarity among physicians regarding the full extent of their obligations to patients. Naturally, in today’s information- and metric-driven environment, healthcare is changing. However, it is important to consider the ramifications and the impact of a changing landscape on the therapeutic patient-provider relationship that defines medicine. Central to the patient-provider relationship is the importance of recognizing and expressly empathizing with the emotional needs of the patient. However, despite the broad consensus that patient-centered care is important and the recognition of empathy affects clinical outcomes, the concept of empathy has actually suffered a decline in the sphere of medical education. The problem arises when incorporating the construct of empathy in medical practice. And this crisis calls for a drastic shift in approach. Rather than learning empathy through cognitive and behavioral practices as traditionally taught in our medical training, empathy should be cultivated uniquely for each individual.

## Review

Patient satisfaction with the healthcare system

Patients are dissatisfied with the current healthcare system, and central to patient frustration is communication. As office and hospital visits become more technical and streamlined for efficiency, less time is spent engaging the patient in meaningful and genuine conversation. These visits result in patient dissatisfaction and ratings of poor communication [1]. Studies by Beckman and Frankel in 1984 and by Marvel and colleagues in 1999 explored how frequently clinicians explicitly asked their patients about their main concerns and how long they listened to their patients’ stories before interrupting. These studies, performed 15 years apart, found that when given an opportunity to tell their story, patients are commonly and quickly interrupted on average within 23 seconds [2,3]. Further, when initiating new medications, physicians often fail to communicate critical elements of medication use, and it was found that only 5% of a 15-minute visit was spent discussing new medications despite the patient’s uncertainty and questions, demonstrating little evidence of shared decision-making [4-6].

Patients want to play an active role in their own medical care. They want to engage in a two-way dialogue, clarify expectations, voice their concerns and ask questions. However, many patients do not see the doctor’s office as a safe place to have those conversations. They are reluctant to ask questions for fear of being perceived as a difficult patient, and they worry about what might be perceived as a challenge to the physician’s authority [7]. These beliefs, compounded with the inherent power differential between the patient and doctor, cause patients to shield themselves and avoid open dialogue with their providers. This attitude is known as the “white-coat silence” phenomenon [8]. Patients entering a new relationship with a provider can experience heightened psychological distress, ranging from feelings of vulnerability as a new patient to fears, situational anxiety, and panic [9].

Healthy relationships

A patient’s relationship with his or her provider requires certain qualities to flourish. Psychologists have long described the qualities and needs required to develop good health and fulfilling human relationships as well as noting the challenges posed by our changing culture. Maslow famously formulated a theory of human motivation, establishing five basic needs that are related to each other and are arranged in a hierarchy. Within this theory, people seek to overcome feelings of loneliness and alienation, and all humans have a need for love, affection, and belongingness [10]. Psychologist Carl Roger noted that people are capable of self-directed growth if they are involved in a specific kind of therapeutic relationship based on humanistic psychology that emphasizes basic tenets of freedom, choice, values, responsibility, autonomy, purpose, and meaning, which run counter to traditional techniques of advice, suggestion, diagnosis, and interpretations [11]. Viktor Frankl, in 1992, suggested that entire generations of doctors were being indoctrinated into what could only lead to a certain cynicism in the study of human existence [12]. He lamented that a kind of schizophrenia rooted in a physiological dysfunction was taking root, leading patients to experience themselves as an object rather than a subject. Rather, he recommended we find meaning in experiential, creational, and attitudinal values (virtues of compassion).

Naturally, our healthcare system should try to emulate the qualities of healthy relationships provided through psychological research. A key step in building a therapeutic relationship is to recognize that some patients may have greater emotional needs and that one approach may not fit all [13,14]. Many patients want to play an active role in their own medical care. They want to engage in a two-way dialogue with their provider, clarify expectations, voice their concerns and ask questions. Patients value affective reassurance through communication that conveys rapport and empathy. They also derive value from cognitive reassurance through clear explanations of their diagnosis and treatment plan [15-21].

Physician discordance with the patient’s needs

Physicians, too, feel the pressures of a changing healthcare landscape. They are under increasing pressure to get the work done in less time, and physicians must balance spending enough time with each patient and staying on schedule. To achieve this balance, physicians may feel forced to interrupt patients and discourage open-ended questions [2]. With continued technological advances, surgical techniques, and new medications, there is a greater range of treatment options to discuss in the shortened time allotted [22]. More pressure is further added with expanded documentation requirements, and an underlying incentive is often placed on documentation extensiveness and, currently, the value of a physician’s work is often judged by how extensively, rather than how pertinently, the documents are recorded [23].

Defining clinical empathy

The concept of patient-centered therapy was derived from Carl Rogers’s theoretical paper published in 1959, “A Theory of Therapy, Personality and Interpersonal Relationships, as Developed in the Client-Centered Framework”. The underlying assumption of his paper was that human beings have an inherent tendency toward growth, development, and optimal functioning which can be cultivated through qualities such as empathy. He defined empathy as the capacity to recognize and, to some extent, share feelings that are being experienced by another [11]. Over the course of 50 years, this has served as the foundation for the development of empathy in healthcare, defined as clinical empathy.

In medicine, we use the mechanism of empathy to facilitate connection and understand our patients. Empathy is a complex process that starts with gaining an insight into the patient’s concerns and feelings of distress, engaging with the patient’s perspective, feeling compassion at the distress of the patient, and then taking action motivated by a desire to alleviate the cause of distress [24].

Empathy effect on healthcare outcome

Under these circumstances, empathy becomes essential to healthcare, and this connection has been demonstrated through numerous studies. Empathy has been linked to medication compliance, better glycemic control, reduced metabolic complications in diabetics, reduced duration of the common cold, and reduced perceptions of pain among numerous other clinical outcomes [25-29]. Further, when patients perceive empathy from physicians, they feel greater satisfaction and empowerment and have less anxiety and distress, which delivers significantly better clinical outcomes [25,30].

Defining the problem

Awareness of the association between empathy and healthcare outcomes has driven The Accreditation Council for Graduate Medical Education (ACGME) and the Association of American Medical Colleges (AAMC) to identify empathy as a key component of medical education and integrate this into a curriculum designed to cultivate empathy [31,32]. However, despite the broad consensus that patient-centered care is important and the recognition of empathy affects clinical outcomes, empathy has actually suffered a decline in the field of medical education [33,34].

The problem arises when incorporating the construct of empathy in medical practice. The medical field increasingly emphasizes the scientific tradition which prioritizes objectivity, technological progress, and certainty. In contrast, empathy seems unpredictable and uncontrollable. Dr. Raul de Velasco explained, “To be empathic is to be 'emotional' rather than objective, a requirement of a scientific attitude. Thus, one who is empathic-rather than 'rational'-will be swayed by the sentiments and suffering of the patient and not be objective enough to make an accurate diagnosis and provide the correct treatment [35]”. This ingrained culture views medical professionals as practitioners who respond to patient suffering with objectivity and detachment [36].

Further evidence in neurobehavioral research has shown that physicians dampen their negative arousal to the pain of others when compared to controls, suggesting mental processing recruits resources away from emotional areas to allow doctors to focus better [37]. Rigid boundaries are set in an attempt to control the interaction and avoid the expression of empathy. The suppression of emotions that arise in death and disability perpetuate coping mechanisms to distance physicians from patient suffering, resulting in a dysfunctional cycle [38].

Traditional empathetic teaching: cognitive-behavioral empathy

To tame the unruly and unpredictable construct of empathy, educators have defined empathy more as a cognitive process taught through cognitive and behavioral skills, termed cognitive-behavioral empathy [39]. This translates into verbal phrases and gestures which serve as surrogates for empathy: “I understand how you feel” or “You seem sad” with a touch on the shoulder. Empathy becomes understood as a means to an end. The result of these cognitive exercises is that empathy becomes a mere intellectual exercise that leads to formulaic and impersonal interactions, which, ironically, lacks real empathy [40]. In clinical practice, cognitive-behavioral empathy leads to a rejection of stories that are unfamiliar to what the physician expects, stigmatizing vulnerable populations [41].

A paradigm shift in empathy teaching

**Figure 1:**
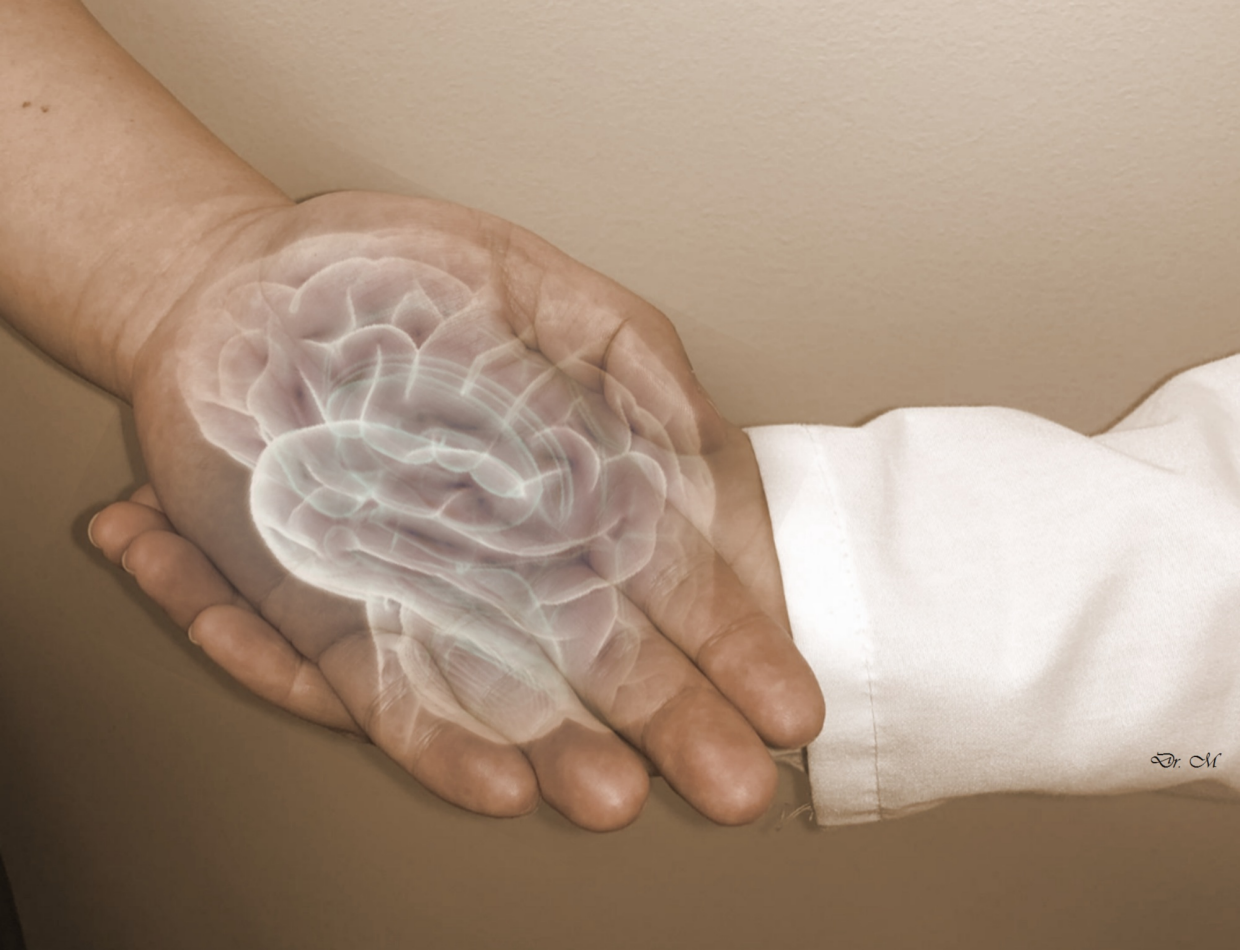
Quality healthcare is dependent on the connection between physicians and patients. Like the patients they treat, physicians must acknowledge and respond to their emotions in contrast to traditional teachings of empathy.

A cultural shift is required to effectively communicate the importance of empathy. Most physicians enter medicine with an inborn sense of compassion, and personal factors such as parents, life experience, and faith have already molded a sense of empathy in them [42]. Thus, these altruistic qualities need to be cultivated rather than empathy being taught by means that promote disconnect. Further, research in the neurosciences has established that empathy consists of not only emotion-sharing and taking the point of view of another as taught by cognitive-behavioral empathy, but also emotion regulation [43]. Empathy involves both the capacity to respond emotionally to the suffering of another as well as the capacity to modulate this experience. This formulation suggests that what is needed is not the ignoring or suppressing of emotion, but its modulation so that it is still recognizably present. Rather than formalized instruction, a cultural shift is needed in medicine to acknowledge the physicians’ needs to process and experience personal emotions [44]. Training in mindfulness, narrative medicine, medical humanities, and reflective writing have all shown empirical promise in recognizing that empathy must be rooted in deeper internal attitudes and behaviors and that suffering really matters [45-50] (Figure 1).

## Conclusions

Empathetic relationships have proven to enhance patient satisfaction and overall patient health outcomes, thereby prompting medical schools, residency programs, and hospitals to promote empathy training. As taught in the traditional cognitive-behavioral sense, empathy becomes a means to an end rather than a genuine interaction. Despite its shortcomings, the traditional teaching of empathy in clinical interactions still prevails, requiring a culture shift in medicine regarding the teaching of empathy. Ideally, empathy should build on the preexisting altruistic qualities that each healthcare professional brings to their career. In other words, the cultivation of empathy should steer clear from superficial, formalized instruction.
